# Self-monitoring and reminder text messages to increase physical activity in colorectal cancer survivors (Smart Pace): a pilot randomized controlled trial

**DOI:** 10.1186/s12885-019-5427-5

**Published:** 2019-03-11

**Authors:** Erin L. Van Blarigan, Hilary Chan, Katherine Van Loon, Stacey A. Kenfield, June M. Chan, Emily Mitchell, Li Zhang, Alan Paciorek, Galen Joseph, Angela Laffan, Chloe E. Atreya, Yoshimi Fukuoka, Christine Miaskowski, Jeffrey A. Meyerhardt, Alan P. Venook

**Affiliations:** 1 Department of Epidemiology and Biostatistics, University of California San Francisco, UCSF Box 0560, 550 16th St. 2nd Floor, San Francisco, CA 94158 USA; 2 Department of Urology, University of California San Francisco, San Francisco, CA USA; 3 School of Medicine, University of California San Francisco, San Francisco, CA USA; 4 Division of Hematology/Oncology, Department of Medicine, University of California San Francisco, San Francisco, CA USA; 5 Helen Diller Family Comprehensive Cancer Center, University of California San Francisco, San Francisco, CA USA; 6 Department of Anthropology, History, and Social Medicine, University of California San Francisco, San Francisco, CA USA; 7 Department of Physiological Nursing, University of California San Francisco, San Francisco, CA USA; 8 Dana-Farber/Partners Cancer Care, Boston, MA USA

**Keywords:** Fitbit, Digital health, Exercise, Accelerometer, Lifestyle, Intervention, Cancer survivor, Colorectal cancer

## Abstract

**Background:**

Over 1.3 million people live with colorectal cancer in the United States. Physical activity is associated with lower risk of colorectal cancer recurrence and mortality. Interventions are needed to increase physical activity in colorectal cancer survivors.

**Methods:**

We conducted a 2-arm non-blinded pilot randomized controlled trial at the University of California, San Francisco among 42 individuals who had completed curative-intent treatment for colorectal cancer to determine the feasibility and acceptability of a 12-week (84 days) physical activity intervention using a Fitbit Flex™ and daily text messages. Participants were randomized 1:1 to receive the intervention with print educational materials or print educational materials alone. We explored the impact of the intervention versus usual care on physical activity using ActiGraph GT3X+ accelerometers pre−/post-intervention.

**Results:**

We screened 406 individuals and randomized 42 to intervention (*n* = 21) or control (*n* = 21) groups. During the 12-week study, the intervention arm wore their Fitbits a median of 74 days [88% of days in study period, interquartile range: 23–83 days] and responded to a median of 34 (out of 46) text messages that asked for a reply (interquartile range: 13–38 text messages). Among the 16 intervention participants who completed the feedback survey, the majority (88%) reported that the intervention motivated them to exercise and that they were satisfied with their experience. No statistically significant difference in change in moderate-to-vigorous physical activity was found from baseline to 12 weeks between arms.

**Conclusion:**

A 12-week physical activity intervention with a Fitbit and text messages was feasible and acceptable among colorectal cancer patients after curative treatment. Larger studies are needed to determine whether the intervention increases physical activity.

**Trial registration:**

Clinicaltrials.gov Identifier NCT02966054. Registered 17 November 2016, retrospectively registered.

**Electronic supplementary material:**

The online version of this article (10.1186/s12885-019-5427-5) contains supplementary material, which is available to authorized users.

## Background

Over 1.3 million individuals currently live with colorectal cancer in the United States (US), representing nearly 10% of all US cancer survivors [1]. Evidence from prospective studies strongly suggests that physical activity after colorectal cancer diagnosis reduces the risk of cancer-specific and overall mortality [2–4]. National guidelines recommend that cancer survivors engage in 150 min per week or more of moderate physical activity [5]. Yet, less than half of colorectal cancer survivors currently meet these recommendations [6]. Interventions that increase physical activity and ultimately improve outcomes among colorectal cancer survivors could provide a great public health benefit.

Digital health tools (e.g., text messaging, physical activity trackers) are a promising approach for increasing physical activity at a lower cost and burden compared to in-person or telephone counseling. A recent systematic review concluded that the majority of randomized controlled trials (RCTs) that included text messaging reported statistically significant improvements in health behaviors (including physical activity) [7]. Most of this research focused on weight loss or management of type II diabetes or cardiovascular disease. A growing number of digital health behavioral interventions have included cancer patients [8], yet colorectal cancer survivors are underrepresented in these studies. In fact, no studies focused on colorectal cancer, and fewer than 30 individuals with colorectal cancer were included in any one study that enrolled multiple cancer types [8].

The benefits of physical activity for colorectal cancer patients is among the most consistent association in the epidemiologic literature of cancer survivorship, yet colorectal cancer survivors are under-represented in lifestyle intervention trials [9, 10]. The lower accrual of colorectal cancer survivors may reflect their lower survival rate (65% 5-y survival vs. 100% for prostate and 89% for breast) as well as the disease’s distinct burden on quality-of-life [11, 12]. Colorectal cancer patients experience unique physical and psychosocial challenges due to their disease, such as living with an ostomy and bowel dysfunction [12]. Consequently, the appropriate content and format of a physical activity intervention among colorectal cancer survivors is unknown, and may differ compared to healthy adult populations or populations of other cancer survivors.

Our long-term goal is to conduct a definitive randomized controlled trial to test the effect of a digital health physical activity intervention on clinical outcomes among colorectal cancer survivors. However, the feasibility and acceptability of a digital health physical activity intervention in these patients is still unknown. Therefore, we developed a digital health intervention, using a Fitbit Flex™ and daily text messages, aimed at increasing physical activity after completion of treatment for colorectal cancer. We conducted a 12-week pilot RCT with 42 colorectal cancer survivors to determine the feasibility and acceptability of the intervention and explore the potential effect of the intervention on accelerometer-measured physical activity.

## Methods

### Study population, consent, and randomization

The University of California, San Francisco (UCSF) Institutional Review Board approved this study. Study data were collected and managed using REDCap electronic data capture tools hosted at UCSF [13].

The target population for this pilot RCT was individuals with non-metastatic colon or rectal cancer who had completed curative therapy. Potentially eligible patients were identified through the Cancer Registry at UCSF or through recruitment in the UCSF Gastrointestinal Oncology Clinic or Gastrointestinal Oncology Survivorship Clinic. Our initial eligibility criteria included: stage II-III colon or rectal cancer, previous completion of cancer-directed therapy ≥3 months and < 2 years prior to enrollment, disease-free status at enrollment, ability to speak and read English, and ability to reliably access the internet and a mobile phone and navigate websites. We excluded individuals with any of 19 specific contraindications to moderate-to-vigorous physical activity (MVPA; e.g., acute myocardial infarction within six months, ongoing unstable angina, uncontrolled cardiac arrhythmia with hemodynamic compromise, active endocarditis) or who were already very active at baseline (defined as engaging in exercise for ≥30 min for ≥5 days a week). Due to initial slow accrual, we expanded the eligibility criteria to include patients with stage I and completely resected stage IV disease and dropped the criteria regarding time after completion of therapy.

**Fig. 1:**
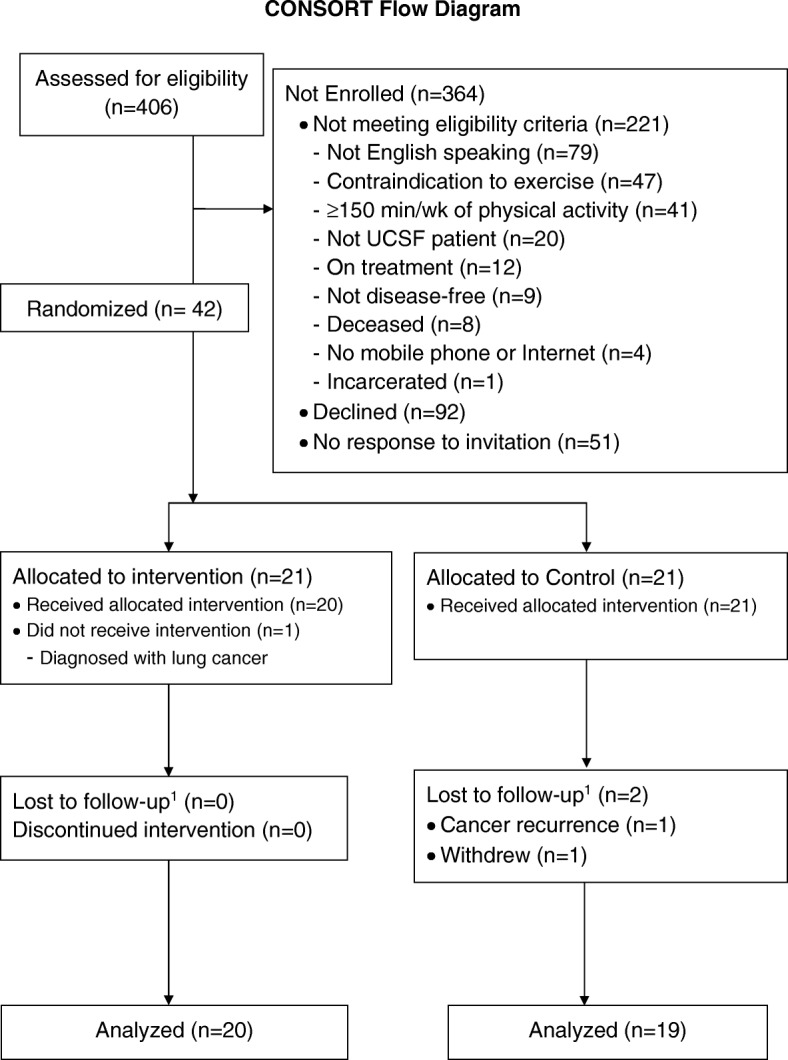
Consort Flow Diagram of a pilot randomized controlled trial of a physical activity intervention among individuals with colon or rectal cancer. Lost to follow-up defined as not completing the 12-week accelerometer assessment

We reviewed the medical records of 406 potentially eligible patients (Fig. 1). Of these, 221 (54%) were ineligible, 92 declined to participate (23%), and 51 never responded to a mailed recruitment letter (13%). The primary reasons for ineligibility included: non-English speaking (79; 36%), contraindications to exercise (47; 21%), and baseline physical activity ≥30 min for ≥5 days a week (exceeding the intervention goal) (41; 19%). Four patients (2%) were excluded for not having a mobile phone.

After screening, informed consent was obtained electronically from all participants included in the study. We randomized 42 individuals who had completed curative treatment for colon or rectal cancer. Participants were randomized 1:1 to intervention or control using a computer-generated randomization scheme developed by a study biostatistician (LZ) who was blinded to group assignment. This scheme was uploaded to REDCap, and a clinical research coordinator utilized the randomization tool in REDCap to obtain the group assignment for each participant.

#### Intervention development

We conducted a semi-structured, in-person, approximately 90 min, group interview with three colorectal cancer survivors (two women and one man) who met our study eligibility criteria to develop our text message content. The participants were 37, 49, and 52 years of age. During the session, we asked participants about their knowledge of physical activity recommendations for cancer survivors, current physical activity behaviors, barriers to physical activity, and gathered feedback on example text messages. While we may not have obtained a diversity of views or saturation of ideas from three individuals, key suggestions from the session included only asking for one-character responses to text messages (e.g., Y for yes) and sending messages either in the morning or evening so they did not arrive during work or family time.

### Intervention description

After completing the baseline assessment and randomization, patients in the intervention arm received print education materials on physical activity after cancer, a Fitbit Flex™, and daily text messages [14–20]. Patients were counseled through the print materials and text messages to build up to 150 min per week of moderate activities, such as walking at a brisk pace, or 75 min per week of vigorous activities, such as jogging or biking, and resistance (strength training) exercise 2–3 times per week.

The Fitbit Flex is a popular, relatively inexpensive, and widely available small wristband that tracks physical activity, including steps, distance, active minutes, and calories burned. The Fitbit Flex provides a valid measure of physical activity [21]. The Fitbit Flex wirelessly synchronizes data to a computer, tablet, or phone and provides participants with feedback through a user-friendly website. With participants’ consent, we accessed their Fitbit data at Weeks 4, 8, and 12 of the intervention period by logging onto their accounts and downloading their data. Participants kept their Fitbit after completion of the study.

Participants received daily text messages to their cellular phone. The text messages were based on the Theory of Planned Behavior and results of the group interview [14]. The message content included information about the benefits of physical activity for colorectal cancer survivors, prompts for goal setting/planning, advice and tips for incorporating activity into daily life, and challenges and quizzes to increase engagement (see Additional file 1 for sample text messages). Text messages were sent once per day in the morning (8a or 9a) or evening (6p or 8p).

Overall, the intervention took approximately 120 min per participant for the research staff to set up and manage over the 12-week intervention period.

### Control arm

After completing the baseline assessment and randomization, control participants received print educational materials about physical activity after cancer. We mailed control participants a Fitbit Flex after completion of the 12-week follow-up accelerometer assessment.

### Measures

#### Feasibility and acceptability assessment

We assessed the feasibility of the intervention through an evaluation of adherence (e.g., Fitbit wear time, response rates to interactive text messages) and attrition (proportion of participants who completed the 12-week follow-up accelerometer assessment). A priori, we stated that the intervention was feasible if we were able to achieve ≥70% adherence and ≤ 20% attrition in the intervention arm. These values were based on the reported adherence for the CanChange trial, a telephone-delivered health behavior intervention in colorectal cancer survivors [22].

We assessed the acceptability of the intervention with an investigator-developed 14-item questionnaire (see Additional file 2) that participants in the intervention arm completed online using REDCap [13]. Participants were asked to what degree they agreed (strongly agree, agree, undecided, disagree, strongly disagree) with four statements about the text messages and one statement about the Fitbit (e.g., “The [text messages, Fitbit] motivated me to exercise.”). For the Fitbit, participants were asked whether they accessed the Fitbit website on a computer and/or via the Fitbit app on their phone; how often they accessed the website and/or app; and whether they would continue to wear the Fitbit after the study ended. Participants were asked how satisfied they were overall with the text messages and Fitbit, separately (i.e., very satisfied, satisfied, neutral, dissatisfied, or very dissatisfied) and provided with an open text box where they could provide any additional feedback.

#### Physical activity assessment

Moderate, moderate-to-vigorous, and vigorous physical activity and daily steps were measured at baseline and 12 weeks in both arms using Actigraph GTX3+ accelerometers [23]. Participants wore the accelerometers around their waist for seven consecutive days. We required at least 3 days of valid wear time, defined as at least 10 h of wear per day [24, 25]. We identified non-wear time using the Troiano 2007 default settings in the ActiLife v6.13.3 software. We used the Troiano 2008 cut-points to identify average min/d engaged in sedentary (0–99 counts per minute), light (100–2019 counts per minute), moderate (2020–5998 counts per minute), and vigorous physical activity (5999 or more counts per minute) [25]. These cut-points were chosen to facilitate comparison with NHANES data, a nationally representative sample. For each patient, we calculated MVPA as the sum of time spent in moderate and vigorous physical activity.

#### Adverse events assessment

At 4, 8, and 12 weeks, participants in both arms were asked to complete a brief online health check-in survey to assess potential adverse events. Participants who did not complete the online survey were first sent a reminder email and were subsequently called by research staff to obtain the information.

### Statistical analysis

To determine if the intervention was feasible, we calculated the proportion of the intervention group who wore their Fitbit Flex N days out of the total 84 study days and the proportion who responded to N text messages out of a total of 46 text messages that asked for a reply. We compared these proportions to the nominal level 70% that we set a priori. For attrition, we calculated the proportion of participants who completed the 12-week accelerometer assessment, and compared that to the nominal level 80%, which was set a priori. To describe physical activity and estimate the effect of the intervention on physical activity, we calculated each patients’ average minutes per day or steps per day based on the 7-day accelerometer at enrollment and 12-weeks. We next calculated each patients’ change in activity levels as the follow-up average minus baseline average, and then for all patients in the intervention arm calculated the mean change and for all patients in the control arm calculated the mean change. We compared the mean change in activity between the two groups using the difference calculated as intervention mean change minus control mean change and 95% confidence interval. Analyses were conducted using SAS v. 9.4.

## Results

We randomized 42 patients with colon or rectal cancer between April 2015 – March 2017 (Fig. 1). Of these 42 randomized patients, 41 participants received their assigned intervention. One patient assigned to the intervention arm was diagnosed with a new primary lung cancer prior to receipt of the intervention and was withdrawn from the study. Follow-up (based on completion and return of the accelerometer at 12-weeks) was 95% complete in the intervention arm and 90% complete in the control arm. The two patients who did not complete the 12-week accelerometer assessment in the control arm withdrew from the study (one due to cancer recurrence and the other due to obtaining her own Fitbit during the study period).

Characteristics of the intervention and control arms at enrollment are presented in Table 1. Both groups were more active according to the Actigraph GT3X+ accelerometer data then compared to their self-reported activity during screening. Overall, the study sample participated in 42 min per day of MVPA at enrollment. In addition, the control arm was more active (51 ± 21 min per day of MVPA and 11,830 ± 4052 steps per day) than the intervention arm (33 ± 18 min per day of MVPA and 9008 ± 3639 steps per day) at enrollment.

**Table 1:** Demographic characteristics, clinical factors, and physical activity at enrollment of 41 colorectal cancer survivors in a pilot randomized controlled trial of a physical activity intervention

Characteristic, mean ± SD or N (%)	Total	Intervention	Control
No. of participants	41	20	21
Age, years	54 ± 11	56 ± 12	54 ± 11
Body mass index, kg/m^2^	28.4 ± 5.9	29.7 ± 7.2	27.1 ± 4.3
Male	17 (41)	8 (40)	9 (43)
Race			
White	30 (73)	14 (70)	16 (76)
African American/Black	1 (2)	1 (5)	0 (0)
Asian	5 (12)	2 (10)	3 (14)
American Indian/Alaskan Native, Native Hawaiian or Pacific Islander, or Other	5 (12)	3 (15)	2 (10)
College degree	38 (93)	17 (85)	21 (100)
Works full-time	26 (63)	14 (70)	12 (57)
Married	20 (49)	9 (45)	11 (52)
Cancer site			
Colon	23 (56)	11 (55)	12 (57)
Rectum	18 (44)	9 (45)	9 (43)
Years since diagnosis	1.5 ± 1.5	1.8 ± 1.9	1.4 ± 1.3
Tumor Stage			
I	8 (20)	4 (20)	4 (19)
II	8 (20)	2 (10)	6 (29)
III	24 (59)	13 (65)	11 (52)
IV	1 (2)	1 (5)	0 (0)
Moderate-to-vigorous physical activity, minutes/day	42 ± 21	33 ± 18	51 ± 21
Steps per day	10,453 ± 4066	9008 ± 3639	11,830 ± 4052

**Fig. 2:**
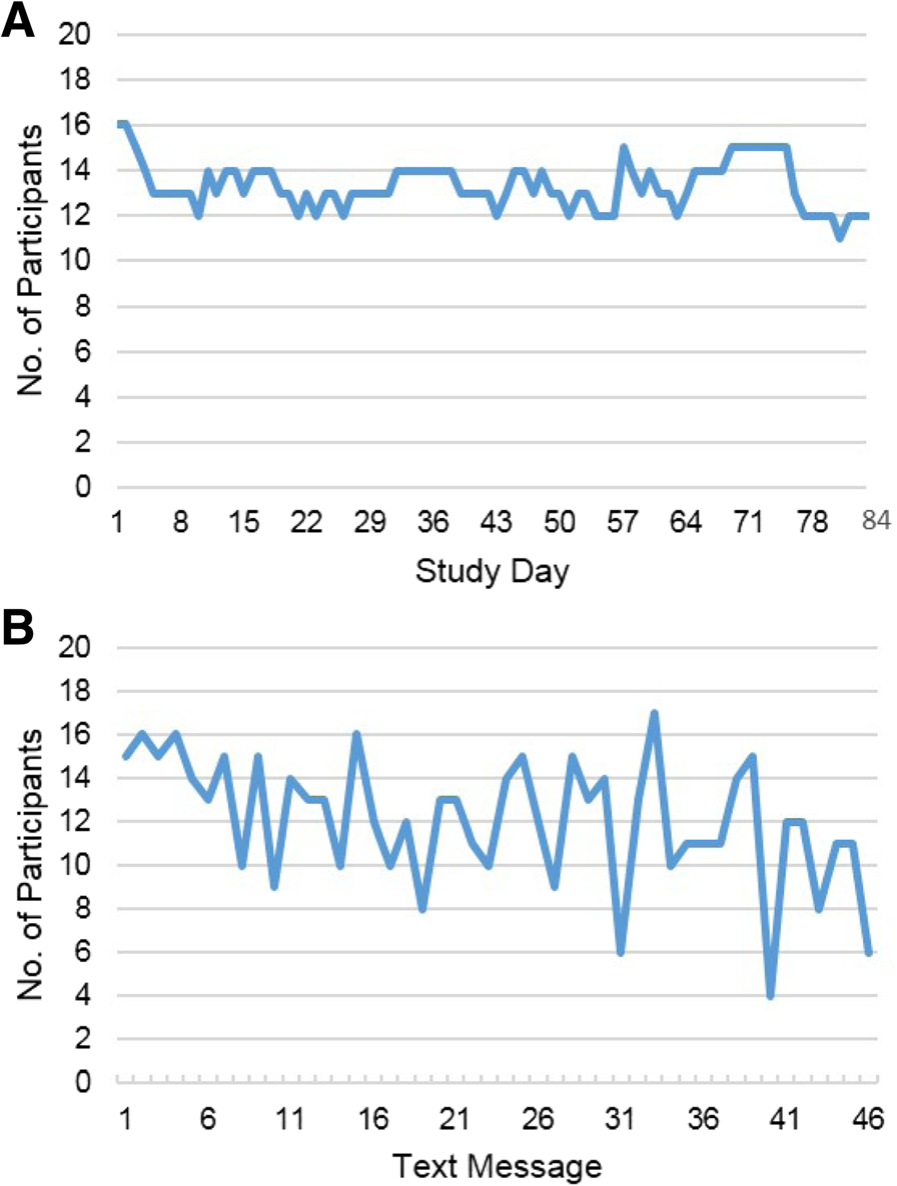
**a** Number of participants who wore their Fitbit by study day. **b** Number of participants who responded to each of the 46 text messages that asked for a response

The intervention was feasible and acceptable to participants. Participants randomized to the intervention arm wore their Fitbits a median of 74 out of 84 days [88% of study days; interquartile range (IQR): 23–83 days] (Fig. 2a). Four participants did not wear their Fitbits (2 had 0 days and 2 had only 1 day of wear), while five participants wore their Fitbits every day.

While participants were overall responsive to the text messages, the response rate decreased over time (Fig. 2b). For example, 15/20 intervention arm participants responded to the first text message, while only 6 responded to the last message that asked for a response. Overall, intervention arm participants responded to a median of 34 out of the 46 text messages that asked for a reply (74%; IQR: 13–38 texts). One participant did not respond to any text messages and one participant responded to every message that asked for a reply.

**Fig. 3:**
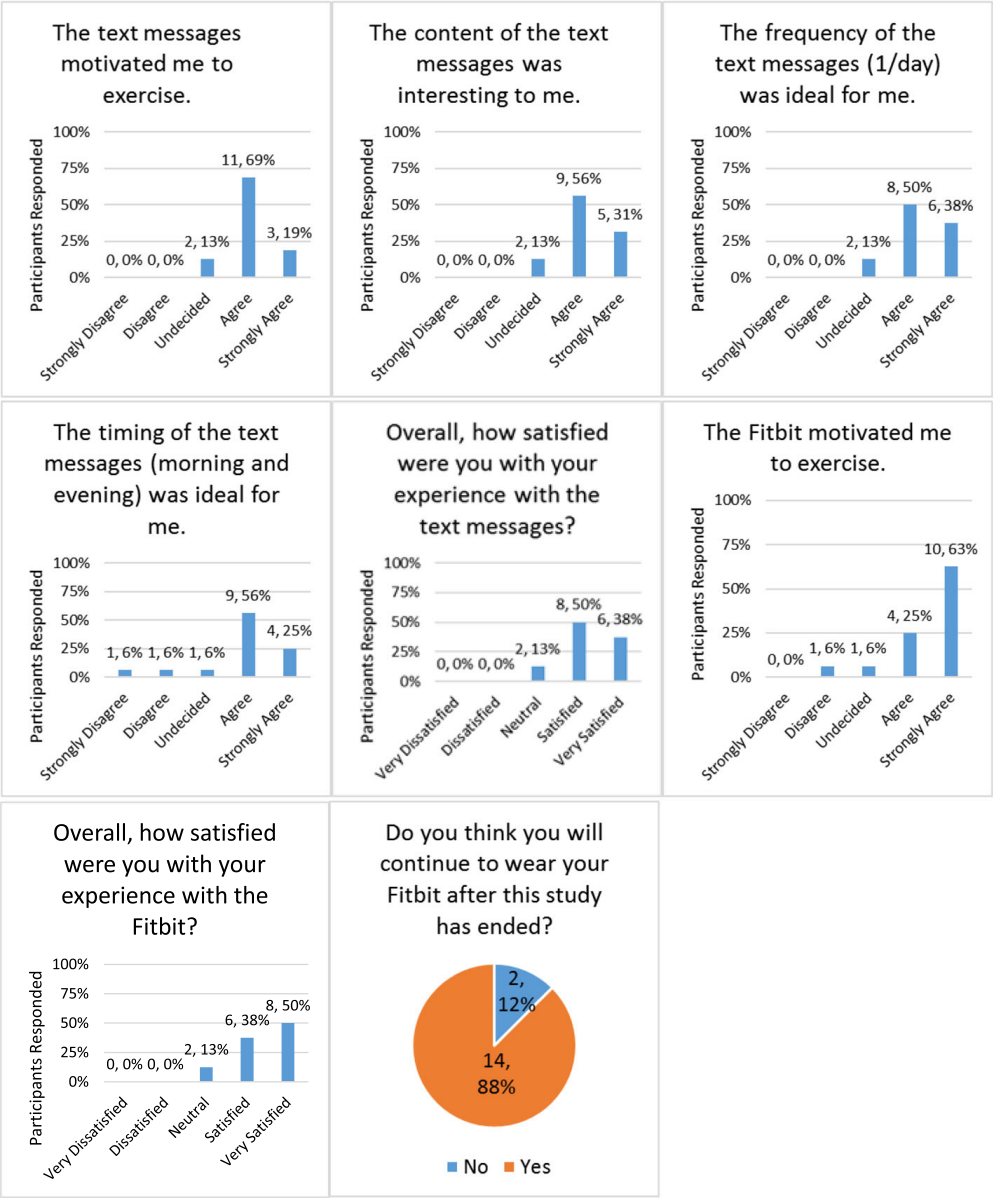
Acceptability of 12-weeks of text messages and a Fitbit Flex to individuals with colorectal cancer. 16 intervention arm participants completed the feedback survey. All values reported are N, %

Consistent with the Fitbit wear time and text message data, participants reported that the intervention was highly acceptable (Fig. 3). Sixteen of the 20 participants in the intervention arm completed the feedback questionnaire. Of these, 14 (88%) agreed that the text messages motivated them to exercise, the content of the text messages was interesting to them, and the frequency of the messages (1 per day) was ideal for them. Thirteen (81%) agreed that the timing of the text messages (morning and evening) was ideal for them. Overall, 88% of participants were satisfied or very satisfied with their experience with the text messages and the Fitbit. Fourteen (88%) of the participants said that they would continue to wear the Fitbit after the study ended.

The mean and standard deviation MVPA, moderate physical activity, vigorous activity, and steps per day measured by accelerometer at enrollment and 12 weeks for participants in the intervention and control groups are in Table 2. The intervention group engaged in a mean of 32.9 min/d of MVPA at enrollment and 46.6 min/d of MVPA at 12-weeks. In contrast, the control group engaged in a mean of 50.8 min/d of MVPA at enrollment and 54.5 min/d of MVPA at 12-weeks. On average, the intervention arm increased their MVPA by 13 min per day more than the control arm (mean difference: 13.1 min per day; 95% CI: -13.5, 39.7) (Table 2).

**Table 2:** Accelerometer-measured physical activity at enrollment and 12 weeks among 41 colorectal cancer survivors participating in a pilot randomized controlled trial of a Fitbit Flex and daily text messages

	Intervention (*n* = 20)		Control (*n* = 19)^a^		
Type of Physical Activity	Baseline Mean ± SD	12 Weeks Mean ± SD	Baseline Mean ± SD	12 Weeks Mean ± SD	Mean difference in change in activity between groups (95% CI)
Moderate-to-vigorous, min/day	32.9 ± 17.9	46.6 ± 48.4	50.8 ± 20.7	54.5 ± 24.9	13.1 (− 13.5, 39.7)
Moderate, min/day	32.1 ± 17.6	43.5 ± 39.0	46.9 ± 18.9	51.7 ± 23.2	9.3 (−12.8, 31.3)
Vigorous, min/day	0.87 ± 0.96	3.1 ± 10.3	3.9 ± 5.2	2.8 ± 3.3	3.8 (− 1.5, 9.1)
Steps per day	9007 ± 3638	10,047 ± 4461	11,829 ± 4052	12,541 ± 5535	649 (− 1581, 2878)

^a^12 week accelerometer data was not available from two participants in the control arm

No serious adverse events occurred during the study. The proportions of participants in the intervention and controls arms who reported a priori*-*identified non-serious adverse events potentially related to physical activity during the study period are in Table 3.

**Table 3:** Non-serious adverse events potentially related to physical activity reported during a 12-week Fitbit and text messaging physical activity intervention among 41 colorectal cancer survivors^a^

	Intervention (*n* = 20)	Control (*n* = 21)
Low back pain, N (%)	6 (30)	8 (38)
Knee pain, N (%)	7 (35)	5 (24)
Inflammation of the joints, arthritis, N (%)	3 (15)	5 (24)
Joint pain, arthralgia, N (%)	3 (15)	1 (5)
Chest pain, N (%)	2 (10)	1 (5)
Shortness of breath, N (%)	2 (10)	2 (10)
Fatigue, N (%)	7 (35)	9 (43)
Leg cramping, N (%)	5 (25)	1 (5)
Muscle pain, N (%)	4 (20)	4 (19)

^a^Participants in both arms were asked to report if they had experienced any of the issues listed in Table 3 at 4, 8, and 12 weeks via an online questionnaire

## Discussion

In this novel pilot RCT, we developed a feasible and acceptable physical activity intervention for colorectal cancer survivors utilizing a physical activity tracker and daily text messages. On average, patients randomized to the intervention arm wore their Fitbit nearly 90% of the time and responded to 74% of text messages that asked for a reply. The vast majority of patients (88%) reported that they were satisfied with their overall experience with the intervention.

Our study had a number of strengths. We developed an interactive text message program to promote physical activity in colorectal cancer survivors. In addition, we enrolled more colorectal cancer survivors than any prior digital health randomized controlled trial, included a racially diverse study sample (27% minorities), and used an accelerometer to measure physical activity at enrollment and 12-weeks.

Limitations of our study included the small sample size; the high level of physical activity, on average, that participants performed prior to enrollment; and a well-educated sample. We used a high cutoff for baseline activity (i.e., 30 min or more on 5 or more days per week based on self-report) when enrolling patients for this pilot study in order to be more inclusive. However, this meant that some patients did not have to change their activity levels very much to meet the intervention goals. Moreover, in contrast to what has been reported by validation studies, participants’ enrollment accelerometer-measured physical activity was even higher than their self-reported activity during screening [26]. Given that our participants had all volunteered for a physical activity intervention study, we postulate that the act of wearing the monitor motivated these individuals to exercise more compared to their usual activity levels [27]. Future studies should focus on less active patients; screen patients activity levels using accelerometers rather than self-report; and consider stratifying on enrollment activity levels to ensure balanced groups. Lastly, 93% of the participants in our study were college educated. Future studies are needed to determine the feasibility and acceptability of this intervention in individuals with different levels of education and socioeconomic status.

While the intervention was determined to be feasible and acceptable, there are aspects that could be improved in future studies. First, we applied pre-determined cut-points for Fitbit wear and text message response rates to assess feasibility. However, the optimal level of physical activity tracker use (e.g., daily, several times a week) to achieve behavior change is not known [28]. Second, we sent daily text messages in the morning or evening based on input from cancer survivors. However, it is not known if this frequency and/or timing of messages optimizes participants’ adherence with the intervention. In addition, participants provided constructive feedback on ways to improve the intervention. One participant suggested that it would be helpful to individualize the text messages based on what participants were recording with their Fitbits: “someone needed to monitor this [the Fitbit data] so other questions or prompts [via text message] could have been given to me.” Future larger studies may be strengthened by having real-time access to participants’ Fitbit data (versus downloading the data periodically) and research staff available to customize messages. Lastly, participants agreed that the Fitbit was a “great motivator,” but one participant had trouble with the band falling off and one participant did not like wearing it at work due to hygiene concerns. Future studies with a larger sample size focused on optimization of the intervention components, including determining whether both components are necessary for behavior change, would be of interest.

## Conclusions

Overall, our pilot study demonstrated that a digital health physical activity intervention is feasible and acceptable to colorectal cancer survivors. A full-scale randomized controlled trial to determine the effect of a digital health physical activity intervention on physical activity in colorectal cancer survivors is warranted.

## Additional files

Additional file 1: Sample text messages in the Smart Pace pilot trial. The first two weeks of text messages sent to participants in the Smart Pace randomized controlled trial. (DOCX 15 kb)

Additional file 2: Smart Pace Feedback Survey. Investigator-developed survey to solicit feedback regarding the Fitbit and text messaging intervention. The survey was self-administered online. (PDF 73 kb)

## Abbreviations

BMI: Body mass index
IQR: Interquartile range
MVPA: Moderate-to-vigorous physical activity
RCT: Randomized controlled trial
UCSF: University of California, San Francisco
US: United States
