# Supplementary material for: Self-monitoring and reminder text messages to increase physical activity in colorectal cancer survivors (Smart Pace): a pilot randomized controlled trial
Source: BMC Cancer. 2019 Mar 11;19:218. doi: 10.1186/s12885-019-5427-5 (PMC6417122; doi:10.1186/s12885-019-5427-5)
Supplement: Supplementary file 1 — Sample text messages in the Smart Pace pilot trial. The first two weeks of text messages sent to participants in the Smart Pace randomized controlled trial. (DOCX 15 kb) [file 12885_2019_5427_MOESM1_ESM.docx]

**Article Title:** Self-monitoring and reminder text messages to increase physical activity in colorectal cancer survivors (Smart Pace): a pilot randomized controlled trial

**Authors**: Erin L. Van Blarigan, Hilary Chan, Katherine Van Loon, Stacey A. Kenfield, June M. Chan, Emily Mitchell, Li Zhang, Alan Paciorek, Galen Joseph, Angela Laffan, Chloe Atreya, Yoshimi Fukuoka, Christine Miaskowski, Jeffrey A. Meyerhardt, Alan P. Venook

**Corresponding Author**: Erin L. Van Blarigan, ScD

Depts. of Epidemiology and Biostatistics, and Urology; University of California, San Francisco

**Address**: UCSF Box 0560, 550 16^th^ St. 2^nd^ Floor, San Francisco, CA 94158

**Phone**: 415-514-1000 ext 13608

**E-mail**: erin.vanblarigan@ucsf.edu

| **The first two weeks of text messages sent to participants in the Smart Pace randomized controlled trial.** | |
| --- | --- |
| **DAY** | **TEXT MESSAGE** |
| 1 | (1/4) Welcome to the Smart Pace Study, brought to you by the UCSF Gastrointestinal Oncology Program and the Helen Diller Comprehensive Cancer Center. |
| 1 | (2/4) Each day or two, you’ll receive a text message. We may ask for a short reply. Text STOP at any time if you no longer want to receive our messages. |
| 1 | (3/4) If you have any questions, call us at [number] or email us at: [email] |
| 1 | (4/4) If you feel ill or get injured, call your doctor or 911. Please text back ‘Y’ to confirm that you received this text. |
| 2 | The American Cancer Society recommends 150 minutes per week of physical activity. Exercise lasting 10 or more minutes counts toward your weekly total! |
| 3 | If you have not been active for a while, start at a comfortable level and add time and intensity gradually. Exercising with a friend may help you get started. |
| 4 | Women who increased their physical activity after colorectal cancer diagnosis had a 49% lower risk of death than women who did not (Meyerhardt JCO 2006). |
| 5 | Wear your Fitbit & sync it with your computer or phone to keep track of your activity. Double tap the small screen to see your progress toward your daily goal. |
| 6 | Good morning! Are you planning to exercise today or did you exercise already today? Text back ‘Y’ or ‘N’. |
| 6 | <if Y or y or yes> Wonderful! |
| 6 | <if N or n or no> If you’re short on time, try to squeeze in exercise by taking the stairs, parking a little further away from your destination, or walking while on the phone. |
| 7 | Take a moment to write down exercise goals for next week, next month, and three months from now. Keep these handy so you can refer to them often. |
| 8 | Schedule time each week to exercise. Take a moment to plan when, where, and with whom you will exercise this week and write it in your calendar. |
| 9 | Are you wearing your Fitbit? Text back Y or N. |
| 9 | <If pt texts back Y, y, yes > Great! |
| 9 | <If pt texts back N,n,no > Put your Fitbit on as soon as you can and wear it every day. Keeping track of your physical activity will help you achieve your exercise goals. |
| 10 | Among >4500 colorectal cancer patients, walking approx. 150 min/wk after diagnosis was associated with a 28% lower risk of death (Schmid *Ann Oncol* 2014). |
| 11 | Take a moment to think about reasons why you have not been physically active in the past. Then write down ideas for how to get past what’s stopping you. |
| 12 | How about a walk after lunch or dinner today? If you can do this, text back ‘Y’. If not, text back ‘N’. |
| 12 | <if patient texts an affirmative response> Great, try to walk for at least 10 minutes! Enjoy your walk! |
| 12 | <if patient texts a negative response> That’s ok, but remember, every little bit counts! The walk doesn’t have to be long – just 10 minutes can improve your health! |
| 13 | Looking for different ways to be active? Try hiking, gardening, golf (walking between holes), bicycling, swimming. Choose something you love, and do it often! |
| 14 | Good morning. Are you planning to exercise today or did you exercise already today? Text back Y or N. |
| 14 | <If pt texts back an affirmative response> Great! |
| 14 | <If pt texts back a negative response> Try to find just 10 minutes today to go for a walk. Remember, a small amount of activity can greatly improve your health. |
