# Supplementary material for: Self-monitoring and reminder text messages to increase physical activity in colorectal cancer survivors (Smart Pace): a pilot randomized controlled trial
Source: BMC Cancer. 2019 Mar 11;19:218. doi: 10.1186/s12885-019-5427-5 (PMC6417122; doi:10.1186/s12885-019-5427-5)
Supplement: Supplementary file 2 — Smart Pace Feedback Survey. Investigator-developed survey to solicit feedback regarding the Fitbit and text messaging intervention. The survey was self-administered online. (PDF 73 kb) [file 12885_2019_5427_MOESM2_ESM.pdf]

SMART PACE FEEDBACK SURVEY

Thank you for participating in the Smart Pace study. We are interested in hearing about your experiences, ideas and opinions about the text messages and Fitbit. Your feedback will help us to develop materials and programs that will be useful to others who have gone through the same experiences as you, so we really appreciate you taking the time to complete this survey.

There are no right or wrong answers to our questions; we are interested in hearing what you think and what experiences you have had. All comments--both positive and negative--are needed.

Think about the text messages you received over the past 3 months.

1. The text messages motivated me to exercise. (strongly agree, agree, undecided, disagree, strongly disagree)
2. The **content** of the text messages was interesting to me. (strongly agree, agree, undecided, disagree, strongly disagree)
   i. If disagree or strongly disagree: How can we improve the content of the text messages?
3. The **frequency** of the text messages (one per day) was ideal for me. (strongly agree, agree, undecided, disagree, strongly disagree)
   i. If disagree or strongly disagree: How can we improve the frequency of the text messages?
4. The **timing** of the text messages during the day (8a, 8p) was ideal for me. (strongly agree, agree, undecided, disagree, strongly disagree)
   i. If disagree or strongly disagree: How can we improve the timing of the text messages?
5. Overall, how satisfied were you with your experience with the text messages? (very satisfied, satisfied, neutral, dissatisfied, very dissatisfied)
6. What feedback do you have about the text messages? Anything you think we should change?

Think about the Fitbit you were asked to wear during the past 3 months.

7. The Fitbit motived me to exercise. (strongly agree, agree, undecided, disagree, strongly disagree)

8. Did you access the Fitbit website on your computer?

    i. How often? (More than once per day, once per day, less than once per day)

9. Did you use the Fitbit app on your phone?

    i. If yes, how often? (More than once per day, once per day, less than once per day)

10. Do you think you will continue to wear your Fitbit after this study has ended?

11. Overall, how satisfied were you with your experience with the Fitbit? (very satisfied, satisfied, neutral, dissatisfied, very dissatisfied)

12. What feedback do you have about the Fitbit? Anything you think we should change?
